# Long Term Effect on Professionals’ Knowledge, Practice and Attitudes towards User Involvement Four Years after Implementing an Organisational Development Plan: A Controlled Study

**DOI:** 10.1371/journal.pone.0150742

**Published:** 2016-03-14

**Authors:** Marit By Rise, Aslak Steinsbekk

**Affiliations:** Department of Public Health and General Practice, Norwegian University of Science and Technology, Trondheim, Norway; University of Kwazulu-Natal, SOUTH AFRICA

## Abstract

**Background:**

Health service organisations are increasingly implementing user involvement initiatives according to requirements from governments, such as user representation in administrational boards, better information to users, and more involvement of the users during treatment. Professionals are vital in all initiatives to enhance user involvement, and initiatives to increase involvement should influence the professionals’ practice and attitudes. The implementation of a development plan intending to enhance user involvement in a mental health hospital in Central Norway had no effect on the professionals after 16 months. The objective was therefore to investigate the long term effect on the professionals’ knowledge, practice and attitudes towards user involvement after four years.

**Methods:**

This was a non-randomized controlled study including professionals from three mental health hospitals in Central Norway. A development plan intended to enhance user participation was implemented in one of the hospitals, including establishing a patient education centre and a user office, purchasing of user expertise, appointing contact professionals for next of kin, and improving of the centre’s information and the professional culture. The professionals at two other hospitals constituted the control group. All professionals were invited to answer the Consumer Participation Questionnaire (CPQ) and additional questions, at a four year interval.

**Results:**

A total of 399 professionals participated (43% response rate). Comparing the changes in the intervention group with the changes in the control group, the results showed that the plan had improved some aspects of the professionals’ knowledge about the user involvement taking place in the hospital. In addition, some parts of the professionals’ practice of providing information to the service users was improved, and the development plan might have raised their awareness about insufficient involvement of next of kin.

**Conclusions:**

This is the first controlled study on the long term effect on professionals from implementing a development plan to enhance user participation in a mental health hospital. Since there was more effect after four years than after 16 months, this study indicates that it takes time before the effect of complex interventions to enhance patient participation in organisations can be detected among the professionals. More long-term studies are thus warranted.

## Background

For several decades user participation in health care has been highly recognized and advocated as a way to improve and ensure the quality of health services [1–4]. There exist different definitions and models for user participation [5–9]. Service users can be involved in decision-making regarding their own treatment or be involved in development of the services; in evaluating the services through patient surveys, participating in decision-making in organisational settings such as committees or boards, or being involved in teaching, training or research processes [5].

To ensure user participation in the health services some Western countries such as Norway have legislated the users’ rights to participate, as well as the services’ duty to involve users [10,11]. This means that health service professionals are obligated to involve service users, both in individual treatment and in provision and development of services. Norwegian governmental regulations and important policy documents show high expectations towards user participation in quality improvement and decision-making; to improve treatment outcome, coordination of services, transparency and patients’ access to information [12]. To comply with the requirements, health service organisations are implementing various initiatives to involve service users in the services [13–15]. However, studies have shown that user involvement is not a matter of course in the health services. Shared decision-making in health care consultations are not widely practiced [13] and although involvement of service users takes place to a certain degree in organizations [16], user participation in health service development is still not the rule [17].

The increased demand on health service organisations to incorporate patient participation in their everyday work [12] might pose challenges, since implementation of change in large organisations is difficult and often fails [18–21]. Furthermore, the implementation of user participation in organisations represents very complex interventions. Complex interventions are described as interventions which include several interacting components, several organisational levels, several expected outcomes, and several behaviours which are required from those who take part [22]. A complex intervention attempts to introduce new patterns of collective action, or to modify existing patterns [23]. There are several factors which determine whether a complex intervention, such as the implementation of user participation, becomes a normalized part of the day-to-day practice [24]. Research investigating the implementation of new and complex initiatives in health service organisations has shown that important factors might be how the initiatives are “enacted” by the professionals and service users [25], how the initiatives are understood by those involved, and who has the responsibility for different parts of the work entailed [25,26]. Focusing only on staff given new tasks without paying attention to their organisational working context may also hinder successful implementation [26].

There exist many facilitators and barriers towards user participation. Involvement of users is highly dependent on the professionals’ knowledge about and attitudes towards involving service users in their daily work. Although service users must be willing and motivated to participate [9,27], professionals are equally vital for bringing about involvement of service users. User participation prerequisites collaboration and a good relationship between service users and professionals [27–29], as well as the processes of dialogue and shared decision-making [9]. Collaboration between service users and professionals can be made difficult if they understand and practice user participation differently [28,30]. In addition, health service professionals are not always equipped with the necessary tools or guidance to be able to involve users [12,31]. Some have also described professionals’ resistance towards user involvement as an important barrier [29,32]. Studies have for instance shown that although professionals agree that service users should be involved in decisions regarding their own treatment, not all agreed that user participation should be extended to service development in organisational settings [33]. Similarly, professionals in some settings still prefer that service users have a consultative role in organisational settings instead of participating in genuine decision-making [32].

However, attitudes towards involvement of users can change over time. Nathan and colleagues investigated professionals’ attitudes before and after the appointing of community representatives in several decision-making committees in the health services [34]. Although professionals felt a lack of knowledge on how to work with representatives before representatives were appointed, one year afterwards, professionals felt that the representatives’ role in the collaboration was clearer, and they had more knowledge on how to work together with the representatives. And more importantly, more professionals felt that the involvement was beneficial and that the health services were ready for such involvement initiatives.

An argument for user involvement, which is used frequently, is that it improves the health services. However, the evidence of effects of user participation is so far mixed [12,14,28,35,36]. Research on initiatives to involve users in organisational settings have shown increased empathy and communication skills in trainees, improvement of information material and interview data, as well as more service user engagement [17]. In addition, involving service users in processes on healthcare improvement processes can change priorities [37]. Several have studied whether implementation of initiatives to involve users can improve the health services by resulting in more systematic integration of patient experiences, increased shared decision-making, higher patient satisfaction, more focus on user involvement, increased collaboration between patient and professional, and improvement of information material and communication, but showed a lack of effect [12,14,28,35,36]. Other expected outcomes in studies have been patient reported knowledge, quality of life, symptom strength, patient adherence [38], anxiety level before surgery, patient understanding and consumer engagement [39]. Overall, the evidence-base for an effect of user participation initiatives in individual treatment and in organizational settings for service development is weak [13, 17, 38–40]. In summary, it is so far an unanswered question whether involving services users on different areas of the health services fills the expectations of improving the services.

Few studies have investigated the long-term effect from user participation initiatives. The reason might be that complex interventions and changes in large organisations often demand a liberal time frame [18,22]. None of the aforementioned studies lasted more than two years [12,14,28,35,36], and it is an open question if this is too little time for measurable changes to settle in the organisations.

In 2009 a comprehensive development plan intended to strengthen user involvement was implemented in a mental health hospital in Central Norway. Implementing multiple initiatives intended to enhance user participation ought to influence the professionals in the organisation. We therefore investigated the effect on professionals’ knowledge, practice, and attitudes towards user involvement 16 months after implementation. The study showed no effects after 16 months [40], and it was discussed whether 16 months might have been too short to see any effects from the development plan. This was strengthened by qualitative interviews exploring the professionals’ experiences with the implementation process where the employees said that the implementation process had taken a long time and that the changes probably were not fully manifested after 16 months [41]. Considering these factors and the lack of studies on long term effects, we wanted to investigate the effect of the development plan after four years.

The aim of this study was therefore to investigate whether implementing an organisational development plan intending to enhance user participation in a mental health hospital had any effect on the professionals’ knowledge, practice, or attitudes towards user participation four years afterwards. Our main interest was whether there was any change in the intervention hospital professionals’ knowledge, practice, or attitudes towards user participation, compared to the professionals in the control hospitals.

## Methods

### Study design and ethics

The present study was a non-randomized controlled study involving professionals from three mental health hospitals in Central Norway. One of the hospitals implemented in 2009 an organisational development plan to enhance user participation (the intervention hospital). The hospital was therefore chosen as the setting for a study to evaluate several aspects of the implementation of the plan. The other two hospitals participated as control group. All professionals working in these hospitals were invited to participate before the implementation and four years afterwards. The study took place from November 2008 to December 2012. The study was approved by the regional committee for medical and health research ethics in Central Norway, the Norwegian Data Inspectorate, and the hospitals’ management. Participants consented to take part in the study by filling out and returning the questionnaire. The ethics committee approved of this consent procedure.

### Setting

The three mental health hospitals (district psychiatric centres) are part of the same university hospital trust. The intervention hospital covers a catchment area of 96.000 persons, with urban and semi-rural areas including parts of a large Norwegian city. The two control hospitals cover catchment areas of 74.000 and 47.000 persons respectively, with urban, semi-rural, and rural areas including parts of the above-mentioned city. The three hospitals provide the same types of service; in-patient treatment (5.4 beds per 10.000 inhabitants), out-patient treatment, and ambulatory services. The intervention hospital was relocated and reorganised in January 2009. Units were merged and co-localised and an ambulatory acute treatment team was established. The reorganisation was based on an over-arching organisational plan focusing on professional development and improvement of collaboration and patient flow. The reorganizational plan also included a development plan for user participation, which was the intervention in this study.

### Intervention

As part of the comprehensive structural reorganization and relocation, the intervention hospital formulated and approved an organisational development plan which was intended to increase user participation. The development plan was formulated by a project group. The group included administrators, health professionals, and user representatives (recruited from mental health user organisations). The group worked from fall 2007 to June 2008. The final plan was approved by the hospital trust in June 2008.

Table 1 gives an overview of the initiatives in the plan. The main aim was to enhance user participation on the individual and system level. The initiatives in the plan were chosen by the project group based on their clinical experience, knowledge about user participation, and the hospital administration’s aim for the health services they provide. We assessed the implementation status in April 2010—fifteen months after the implementation started—and found that most of the initiatives were completed (Table 1). This information was collected through interviews with key professionals and documents produced by the hospital. The implementation status in April 2010 was confirmed by the hospital’s management.

**Table 1:** Initiatives in the development plan.

Planned initiatives in development plan sanctioned in June 2008 and planned implemented from January 2009	Status for implementation in April 2010
Establishing a patient education centre	A patient education centre was established in November 2009, and employed two persons. A user representative participated in the planning and starting of the centre, and representatives partake in the daily work.
Establishing an office run by users where various user representatives shall be available to the users of the centre	An office and information centre for users was established in January 2010. The office provides information material, telephone and Internet for patients and next of kin. Two user organisations and representatives from the regional labour and welfare administration use the office weekly.
Purchasing user expertise up to 17.5 hours per week	The centre’s budget allows for buying up to 17.5 hours of user expertise per week, but normally buys 10–12 hours per month. A user representative is employed 20% for the research project on self-administered places/beds.
Establishing a strategy for education of user representatives	Not implemented. Education of user representatives has been assigned to the user organisations.
Appointing contact personnel for next of kin in each section	In March 2009 one personnel from each unit has been appointed contact person for next of kin.
Allowing money in the budget for patient education	Money for patient education have since January 2009 been a part of the patient education centre’s working budget.
Tentative proceedings with places/beds administered by the patients themselves	A randomized controlled trial on places/beds administered by patients was started in May 2010. One user representative is participating in the steering committee, and two in the research group. User expertise equivalent to 20% employment is bought during this study.
Improving the centre’s communication and information materials	A group was established before relocation to evaluate and suggest measures to improve the centre’s communication and information materials. The work in this group stopped after a few meetings. Outwards communication has been discussed at several staff meetings during 2009 and 2010.
Formulate and implement a strategy for quality assurance of attitudes and culture among the personnel	Tentative plans were discussed with user representatives in spring 2009. A philosopher was temporarily employed during the fall 2009. He conducted group sessions with health personnel to discuss attitudes towards user participation. The work stopped in 2009. The implementation group (administrators, health personnel and user representatives) discussed attitudes and culture at 6–8 meetings during the implementation process.
Implementing a web based system (Sampro) for collaborating and coordinating individual plans and individual education plans for patients.	An educational course led by an external course supervisor was held for 4 patients and their therapists in April 2010. In one of the in-patient units therapist have received training in using the system, and patients are continuously offered to use this system.
Informing patients; in general about the centre, about their right to change therapist, and about setting treatment goals	Information has been discussed at several meetings in the executive group, but no concrete initiatives have been planned or implemented.
Tentative proceedings with using Client Directed Outcome Informed therapy in out-patient sessions.	A research trial on Client Directed Outcome Informed therapy in out-patient sessions started in February 2010, and is currently running.
(Not in development plan)	The patient education centre reviewed each unit’s work with patient education from January 2010, and decided to appoint one contact person for patient education per unit. Per April 2010 6 out of 8 units had contact persons.
(Not in development plan)	To ensure identification of and care for in-patients’ children a group in charge was appointed in January 2010.
(Not in development plan)	All in-patient units conduct regular “house meetings” where patients are encouraged to raise issues which are subsequently discussed in management meetings.
(Not in development plan)	Patients and users are represented in the panel overseeing the quality of the services, and are participating in the processes of introducing new service initiatives.

The development plan was implemented from January 2009. The hospital manager was in charge of the implementation process, and the everyday executive responsibility was ensured by one of three unit managers at the hospital. An implementation group consisting of the unit manager, several administrators, health professionals, and user representatives recruited from mental health user organisations was established in August 2009 to supervise and follow up the implementation. The group had six meetings until January 2010 when the group was dissolved.

### Participants

All employees including psychiatrists, nurses, psychologists, other health and social workers, and administrative professionals in the three mental health hospitals were invited to participate in this study. They were identified from lists including all paid employees registered by the hospitals’ administrative offices the current month. To maintain anonymity and to invite all employees at both measurement points, we did not match the employees participating at the different time points. Although several employees probably participated at both time points, the group of employees answering the questionnaire were not exactly the same at the first and second time of measurement. They were thus treated and analysed as four independent samples. The differences in results from baseline to follow-up in sample 1 and sample 2 was compared with the differences in results from baseline to follow-up in sample 3 and 4.

### Data collection

All employees at the intervention hospital were invited to fill out a paper questionnaire in December 2008 (before the implementation of the development plan) and in December 2012 (four years afterwards). All employees at the control hospitals were also invited to fill out the same questionnaire twice with a four year interval during the same period. All invitations to fill out questionnaires were sent by post to all employees’ private address with pre-paid return envelopes which were returned directly to the researchers. One reminder was sent in December 2008. No reminders were sent in December 2012.

### Outcome measures

To measure knowledge, practice, and attitudes towards user participation among the professionals the Consumer Participation Questionnaire (CPQ) was used [42]. The questionnaire was translated to Norwegian for this study [40]. The original CPQ questionnaire includes 20 questions. To include question about user organisations, question no. 8 in the original questionnaire (Does your agency solicit user input for planning of mental health service?) was supplemented with a new question no. 8a (Does your agency solicit input from user organisations for planning of mental health service?). Question no. 14 in the original (Should users be involved in the evaluation and diagnosis of their presenting problems?) was split in two questions to ensure interpretable results (no. 14 and 14a). Answers to question no. 2 were dependent on the answer to no. 1 and the question was omitted in the analyses for this paper.

We also added 8 questions to ensure that aspects of the professionals’ views and practices regarding user participation not asked about in CPQ were included. These questions were formulated, discussed, and refined during meetings in the research group. The questionnaire used in this study thus included a total of 29 questions.

The questions were organised into three thematic areas for this study: Eleven of the 29 questions measured professionals’ knowledge on user participation, seven measured practice, and 11 measured attitudes.

### Statistical analysis

The results from the two control hospitals were combined. Pearson’s chi square tests were used to identify any differences in proportions within the two groups at the two points of measurement. For each questionnaire item, differences in proportions between baseline sample and follow-up sample were calculated using binary logistic regression. We conducted analyses for the intervention hospital and the control hospitals, respectively. Demographic variables with trends for difference (p<0.1) within each group (intervention group; current position, control group; gender and current position, Table 2) were added to the time of measurement variable in the regression model. To compare the odds ratio (OR) in the intervention group to the OR in the control group we used a test of proportion [43] to calculate ratio odds ratios (ROR). A ratio odds ratio tells us whether there are any differences between the changes in two different groups. A test of proportions is used to compare two estimated quantities, such as means or proportions (in this case odds ratios) of the same quantity in two independent samples. All statistical analysis was done with SPSS 21.0 for Windows (IBM Corp., Armunk, NY) with a significance level of 5% (p<0.05).

**Table 2:** Demographic variables—comparison of proportions at baseline and four years afterwards. Numbers are percentages of total N for each sample unless otherwise stated.

*Variable*	*Intervention group*			*Control group*		
	Baseline % N = 89*	4 years % N = 84*	*p-value*^†^	Baseline % N = 133*	4 years % N = 93*	*p-value*^†^
*Female*	72.7	71.4	0.849	87.0	77.4	0.064
*Current position*			0.129			0.281
- Nurse	36.4	26.2	(0.151)	28.5	22.3	(0.302)
- Medical doctor	4.5	9.5	(0.200)	7.7	12.8	(0.208)
- Psychologist	21.6	35.7	(0.040)	13.8	22.3	(0.098)
- Health/welfare worker	25.0	16.7	(0.179)	37.7	28.7	(0.162)
- Administrative /Other	12.5	11.9	(0.905)	12.3	13.8	(0.738)
*Leadership responsibility*	12.5	8.5	0.401	7.7	6.4	0.707
*Category of patients working with*			0.795			0.626
- In-patients	29.5	26.2	(0.624)	49.6	42.6	(0.295)
- Out-patients	52.3	59.5	(0.338)	32.8	39.4	(0.312)
- Both	11.4	8.3	(0.506)	12.2	10.6	(0.715)
- Not working with patients	6.8	6.0	(0.817)	5.3	7.4	(0.519)
*No of years worked in this unit*. *Mean(SD)*	6.4 (6.3)	5.6 (5.7)	0.361^‡^	6.8 (6.2)	7.0 (6.5)	0.856^‡^

* The N in the four samples varied for each question due to missing answers on the variables (0.8%–2.5%).

^†^ P-value calculated using Pearson’s chi square.

^‡^ P-value calculated using independent samples t-test.

## Results

Eighty-nine of 184 (48%) members of professionals responded at the intervention hospital at baseline (sample 1), and 84 of 226 (37%) responded 4 years later (sample 2). At the control hospitals, 133 of 221 (60%) professionals responded at baseline (sample 3), while 93 of 297 (31%) responded after 4 years (sample 4).

The total sample (N = 399) included 78.3% females, 28.3% nurses, 28.3% health/social workers, 22.2% psychologists, 12.6% administrative/others, and 8.6% medical doctors, while 8.6% had leadership responsibility. Of the participants 38.5% worked with in-patients, 44.3% with out-patients, 10.8% worked with both groups, and 6.4% did not work therapeutically with patients. The participants at baseline had worked between three months and 29 years in the unit with a mean of 6.7 years (SD 6.2) and a median of 5 years. The participants at follow-up had worked between two months and 31 years with a mean of 6.3 years (SD 6.1) and a median of 5.0.

Differences in demographics between baseline and follow-up within each of the two groups are described in Table 2. In the intervention group there were significantly more psychologists in the sample at follow-up compared to baseline (p = 0.040). In the control group there was a trend for more psychologists (p = 0.098) and more females (p = 0.064) in the follow-up sample, compared to baseline. These variables were added to the binary logistic regression models for the groups respectively and were thus controlled for when comparing the changes within and between the groups.

The proportions of answers in the intervention group and control group were not systematically different at baseline. (Proportions of answers at baseline given in Table 3, analysis of baseline comparison is not shown).

**Table 3:** Personnel’s knowledge, practice, and attitudes at baseline and four years afterwards—intervention and control group. Numbers are percentages of total N for each sample unless otherwise stated.

	*Intervention*			*Control*		
Knowledge	*Baseline % N = 89*^1^	*4 years % N = 84*^1^	*p-value*^†^	*Baseline% N = 133*^1^	*4 years % N = 93*^1^	*p-value*^†^
*1*. *Does your unit have a complaints procedure for clients*? (% yes)	47.7%	72.6%	0.001**	66.7%	72.0%	0.391
*5*. *Have you heard or read anything about consumer involvement and participation in the provision of mental health services*? (% yes)	91.0%	92.7%	0.690	88.6%	88.2%	0.915
*8a*. *Does your unit solicit input from users for the planning of mental health services*? (% yes)	69.7%	70.2%	0.934	74.2%	60.9%	0.034**
*8b*. ^#^ *Does your unit solicit input from user organisation for the planning of mental health services*? (% yes)	47.2%	58.3%	0.142	36.4%	45.7%	0.156
*9*. *Does your unit routinely conduct consumer satisfaction service on the service it offers*? (% yes)	34.1%	36.9%	0.700	37.9%	41.9%	0.540
*10*. *Are consumers involved in the hiring decisions of your unit’s staff*? *(% yes)* ^††^	0%	1.2%	0.299	0%	1.1%	0.232
*11*. *Are consumers invited to participate in staff training meetings at your agency*? (% yes)	3.4%	20.5%	<0.001**	6.8%	14.0%	0.075*
*12*. *Has your unit ever asked clients to act as teachers at staff training events*? (% yes)	7.9%	16.7%	0.076*	16.8%	27.7%	0.050*
*13*. *Does your unit sponsor events/forums that educate consumers about their rights and entitlements*? (% yes)	37.1%	67.9%	<0.001**	61.1%	46.8%	0.034**
*21*. ^#^ *Does the unit have a users’ committee*? (% yes)	28.1%	44.0%	0.029**	56.5%	51.6%	0.470
*22*. ^#^ *Does the unit have representatives or spokespersons on behalf of the users*? (% yes)	22.5%	30.1%	0.254	51.9%	53.8%	0.784
**Practice**						
*3*. *Are clients told they have a right to see and/or correct their records*? (% yes)	58.4%	71.4%	0.074*	58.3%	65.6%	0.271
*4*. *Are clients informed about the facts about confidentiality and privacy regarding information contained in those records*? (% yes)	79.8%	92.9%	0.013**	84.8%	78.7%	0.234
*7*. *Do you tell clients what goals are intended to be accomplished by the treatment*? (% yes)	81.2%	84.0%	0.638	89.1%	88.9%	0.968
*25*. ^#^ *Do you have enough time to ensure users’ participation*? (% yes)	59.3%	77.5%	0.013**	67.8%	84.3%	0.007**
*26*. ^#^ *In your opinion*, *are next of kin in general sufficiently involved*? (% yes)	30.1%	32.5%	0.743	25.2%	53.0%	<0.001**
*27*. ^#^ *How would you describe the collaboration with next of kin in general*?(% very good/quite good)	44.6%	52.4%	0.312	42.3%	58.0%	0.023**
*28*. ^#^ *Do you inform users about relevant self-help groups and user organisations*? (% yes)	85.4%	83.8%	0.776	78.4%	73.9%	0.442
**Attitudes**						
*6*. *In most cases*, *where does the responsibility for deciding the goals of treatment usually lie*? (% entirely/mostly the client)	6.8%	8.3%	0.707	12.0%	12.9%	0.845
*14*. *Should clients be involved in the evaluation of their presenting problems*? (% always/usually)	87.5%	91.6%	0.387	90.0%	94.6%	0.220
*14a*. *Should clients be involved in the diagnosis of their presenting problems*? (% always/usually)	80.9%	77.4%	0.569	66.4%	69.9%	0.583
*15*. *In your opinion*, *should clients contribute to the writing of their notes and records*? (% yes)	43.7%	35.7%	0.287	42.5%	39.3%	0.639
*16*. *In your opinion*, *should clients be involved in the planning of their own treatment*? (% yes)	97.7%	97.6%	0.962	97.0%	97.8%	0.687
*17*. *How would mental health service change if consumers were employed by that service*? (% improve)	71.6%	69.1%	0.731	56.2%	57.7%	0.835
*19*. *How would mental health service change if consumers were involved in the planning and/or delivery of those services*? (% improve)	90.7%	87.8%	0.545	81.7%	77.9%	0.491
*23*. ^#^ *How would you describe the unit’s general attitude towards user participation*? (% quite good/very good)	32.2%	53.0%	0.006**	61.8%	68.1%	0.335
*24*. ^#^ *In your opinion*, *do users understand the information you give about their illnesses and treatment opportunities*? (% yes)	98.8%	92.7%	0.058*	95.9%	96.6%	0.800
*18*. *In your opinion*, *what are the most important reasons when users of mental health care don’t want to be involved*?:						
- Too vulnerable (% yes)	36.0%	40.5%	0.541	42.9%	34.0%	0.180
- Lacking in self-confidence (% yes)	59.6%	52.4%	0.342	60.2%	50.0%	0.129
- Lacking in ability or knowledge (% yes)	12.4%	19.0%	0.226	14.3%	10.6%	0.417
- Lacking in motivation (% yes)	51.7%	41.7%	0.187	42.1%	47.9%	0.389
- Lack of trust in the ability of the services to provide help (% yes)	38.2%	33.3%	0.504	24.8%	23.4%	0.807
- Not wanting to have any further contact after getting better (% yes)	44.9%	45.2%	0.969	37.6%	42.6%	0.452
- Other reasons (% yes)	16.9%	15.5%	0.806	18.0%	13.8%	0.397
*20*. *In your opinion*, *if users were involved in planning and/or carrying out the mental health service*, *how would the service develop*?:						
- Upgrading of services and delivery (% yes)	73.0%	60.7%	0.085*	56.4%	52.1%	0.525
- Less burnout and stress for providers of those services (% yes)	11.2%	16.7%	0.302	9.0%	5.3%	0.296
- More chance that users would benefit from those services the first time round (% yes)	86.5%	72.6%	0.023**	71.4%	61.7%	0.124
- Less chance of the “revolving door” syndrome, where users keep returning with the hope of finding help (% yes)	27.0%	34.5%	0.281	27.1%	22.3%	0.419
- Downgrading of services and delivery (% yes)	1.1%	4.8%	0.153	5.3%	1.1%	0.091*
- More burnout and stress for the providers of those services (% yes)	3.4%	6.0%	0.419	6.8%	6.4%	0.909
- That users would only be regarded as tokens by the professionals (% yes)	9.0%	7.1%	0.656	7.5%	11.7%	0.284
- That users would not understand the language used, and therefore find it difficult to give any input (% yes)	3.4%	2.4%	0.698	8.3%	7.4%	0.821
- Other developments (% yes)	7.9%	14.3%	0.177	9.0%	7.4%	0.673

^1^ N is the number of participants who returned completed questionnaires. The N in the four samples varied for each question due to missing answers on the variables (0%–11%).

^*#*^ Questions marked ^#^ were added to the Consumer Participation Questionnaire (CPQ) in this study.

* p<0.1,

** p<0.05,

^†^ p-value calculated using Pearson’s chi square test.

^††^Question no. 10: Numbers too small for logistic regression analysis, results only given in Table 3.

### Changes within the study groups

Table 3 shows the distribution of answers and tests of difference in proportions between baseline to follow-up after 4 years for the intervention group and control group. In question no. 10 “Are service users involved in the hiring decisions of the unit’s staff?” the proportion of respondents answering “yes” (2 participants) was too small to make any meaningful comparisons. The question was therefore omitted from the analyses. Table 4 (first two columns) shows the comparison of changes within the study groups for variables with p≤0.2.

**Table 4:** Comparison of changes within and between the groups. The table only show variables with p≤0.2. ROR>1.0 favours intervention. AdjOR >1.0 favours increase since baseline.

	*WITHIN (changes within each group from baseline to four years)*				*BETWEEN (comparison of the groups)*	
	*Intervention N = 173*^1^		*Control N = 226*^1^		*Intervention vs*. *Control*	
*Variable*	*AdjOR (95% CI)*	*p-value*^†^	*AdjOR (95% CI)*	*p-value*^†^	*Ratio OR (95% CI)*	*p-value*^†^
**Knowledge**						
*1*. *Does your unit have a complaints procedure for clients*? *(% yes)*	2.97 (1.57–5.63)	0.001**	1.45 (0.80–2.63)	0.222	2.0 (0.9–4.9)	0.107
*8a*. *Does your unit solicit input from users for the planning of mental health services*? (% yes)	1.05 (0.55–2.02)	0.879	0.54 (0.30–0.96)	0.036**	1.9 (0.8–4.7)	0.135
*8b*. ^#^ *Does your unit solicit input from user organisation for the planning of mental health services*? (% yes)	1.60 (0.86–2.93)	0.127	1.50 (0.87–2.60)	0.148	1.1 (0.5–2.4)	0.878
*11*. *Are users invited to participate in staff training meetings at your unit*? (% yes)	11.27 (2.50–50.88)	0.002**	2.15 (0.86–5.39)	0.103	5.2 (0.9–30.6)	0.066
*12*. *Has your unit ever asked users to act as teachers at staff training events*? (% yes)	2.74 (1.00–7.52)	0.050*	1.95 (1.00–3.78)	0.049**	1.4 (0.4–4.7)	0.581
*13*. *Does your unit sponsor events/forums that educate consumers about their rights and entitlements*? *(% yes)*	3.70 (1.97–6.96)	<0.001**	0.57 (0.33–0.98)	0.043**	6.5 (2.8–14.9)	<0.001**
*21*. ^#^ *Does the unit have a users’ committee*? (% yes)	1.98 (1.05–3.73)	0.036**	0.88 (0.51–1.51)	0.644	2.3 (1.0–5.2)	0.057
**Practice**						
*3*. *Are clients told they have a right to see and/or correct their records*? *(% yes)*	1.73 (0.91–3.27)	0.091*	1.49 (0.85–2.62)	0.168	1.2 (0.5–2.7)	0.731
*4*. *Are clients informed about the facts about confidentiality and privacy regarding information contained in their records*? *(% yes)*	3.42 (1.28–9.13)	0.014**	0.72 (0.35–1.44)	0.349	4.8 (1.4–15.9)	0.012**
*25*. ^#^ *Do you have enough time to ensure users’ participation*? (% yes)	2.36 (1.19–4.70)	0.014**	2.45 (1.21–4.93)	0.012**	1.0 (0.4–2.6)	0.940
*26*. ^#^ *In your opinion*, *are next of kin in general sufficiently involved*? (% yes)	1.07 (0.54–2.11)	0.843	3.27 (1.80–5.96)	<0.001**	0.3 (0.1–0.8)	0.016**
*27*. ^#^ *How would you describe the collaboration with next of kin in general*? (% very good/quite good)	1.33 (0.72–2.48)	0.362	2.07 (1.17–3.68)	0.013**	0.6 (0.3–1.5)	0.304
**Attitudes**						
*23*. ^#^ *How would you describe the unit’s general attitude towards user participation*? (% quite good/very good)	2.44 (1.30–4.58)	0.006**	1.50 (0.84–2.69)	0.172	1.6 (0.7–3.8)	0.266
*24*. ^#^ *In your opinion*, *do users understand the information you give about their illnesses and treatment opportunities*? (% yes)	0.13 (0.02–1.16)	0.067*	0.87 (0.19–4.02)	0.858	0.1 (0.0–1.9)	0.142
*18*. *In your opinion*, *what are the most important reasons when users of mental health care don’t want to be involved*?						
- Too vulnerable (% yes)	1.26 (0.68–2.33)	0.470	0.72 (0.41–1.25)	0.243	1.8 (0.8–4.0)	0.187
- Lacking in self-confidence (% yes)	0.77 (0.42–1.40)	0.765	0.66 (0.38–1.13)	0.131	1.2 (0.5–2.6)	0.710
- Lacking in ability or knowledge (% yes)	1.92 (0.81–4.55)	0.141	0.69 (0.30–1.57)	0.374	2.8 (0.8–9.2)	0.093*
- Lacking in motivation (% yes)	0.69 (0.38–1.26)	0.227	1.26 (0.74–2.16)	0.402	0.5 (0.2–1.2)	0.142
*20*. *In your opinion*, *how would the service develop if users were involved in planning and/or carrying out the mental health service*?						
- Upgrading of services and delivery (% yes)	0.58 (0.31–1.11)	0.101	0.82 (0.48–1.41)	0.472	0.7 (0.3–1.6)	0.416
- Less burnout and stress (% yes)	1.60 (0.66–3.85)	0.295	0.60 (0.20–1.79)	0.357	2.7 (0.7–10.9)	0.172
- More chance that users would benefit from those services the first time round (% yes)	0.41 (0.19–0.91)	0.028**	0.65 (0.37–1.15)	0.138	0.6 (0.2–1.7)	0.350
- Less chance of a revolving door syndrome (% yes)	1.49 (0.77–2.86)	0.237	0.82 (0.44–1.54)	0.538	1.8 (0.7–4.5)	0.197
- Downgrading of services and delivery (% yes)	4.59 (0.50–42.34)	0.179	0.19 (0.02–1.60)	0.126	24.2 (1.1–546.4)	0.045**
- Other developments (% yes)	1.92 (0.72–5.14)	0.195	0.79 (0.30–2.12)	0.645	2.4 (0.6–9.7)	0.209

^1^ N is the no of participants who returned completed questionnaires. The N in the four samples varied for each question due to missing answers on the variables (0%–11%).

* p-value < 0.1

** p-value < 0.05

^#^ Questions marked ^#^ are added to the Consumer Participation Questionnaire (CPQ) in this study.

^†^ p-value calculated using logistic regression and test of proportions.

#### Knowledge about user participation

At the intervention hospital there was significant change at a 0.05 level in five out of eleven questions; No 1 “Does your unit have a complaints procedure for clients?” (AdjOR = 2.97, CI = 1.57–5.63, p = 0.001), no. 11 “Are users invited to participate in staff training meetings at your unit?” (AdjOR = 11.27, CI = 2.50–50.88, p = 0.002), no. 12 “Has your unit ever asked users to act as teachers at staff training events?” (AdjOR = 2.74, CI = 1.00–7.52, p = 0.05), no. 13 “Does your unit sponsor events/forums that educate consumers about their rights and entitlements?” (AdjOR = 3.7, CI = 1.97–6.96, p<0.001) and no. 21 “Does the unit have a users’ committee?” (AdjOR = 1.98, CI = 1.05–3.73, p = 0.036).

At the control hospitals there was significant change at a 0.05 level in three out of eleven questions; No 8a “Does your unit solicit input from users for the planning of mental health services?” (AdjOR = 0.54, CI = 0.30–0.96, p = 0.036), no 12 “Has your unit ever asked users to act as teachers at staff training events?” (AdjOR 1.95, CI = 1.00–3.78, p = 0.049) and no 13. “Does your unit sponsor events/forums that educate consumers about their rights and entitlements?” (AdjOR = 0.57, CI = 0.33–0.98, p = 0.043). There were no trends for change (p<0.1) in either of the groups.

#### Practice of user participation

There were significant changes on the 0.05 level on two out of seven questions in the intervention group; No. 4 “Are clients informed about the facts about confidentiality and privacy regarding information contained in their records?” (AdjOR = 3.42, CI = 1.28–9.13, p = 0.014) and no. 25 “Do you have enough time to ensure users’ participation?” (AdjOR = 2.36, CI = 1.19–4.70, p = 0.014). There was a trend for change on question no 3 “Are clients told they have a right to see and/or correct their records? (AdjOR = 1.73, CI = 0.91–3.27, p = 0.091).

In the control group there were significant changes in three out of five questions; No 25 (AdjOR = 2.45, CI = 1.21–4.93, p = 0.012), no. 26 “In your opinion, are next of kin in general sufficiently involved?” (AdjOR = 3.27, CI = 1.80–5.96, p<0.001) and no. 27 “How would you describe the collaboration with next of kin in general?” (AdjOR = 2.07, CI = 1.17–3.68, p = 0.013). There were no questions with a trend for change in the control group.

#### Attitudes towards user participation

In the intervention hospitals there were significant changes from baseline to follow-up regarding the professionals’ attitudes towards user participation at one of the 9 questions; No 23 “How would you describe the unit’s general attitude towards user participation?” (AdjOR = 2.44, CI = 1.30–4.58, p = 0.006). There was a trend for change within the intervention group in one question; No 24 “In your opinion, do users understand the information you give about their illnesses and treatment opportunities?” (AdjOR = 0.13, CI = 0.02–1.16, p = 0.067).

There were no significant changes or any trends for change in the control group.

### Comparison between the groups

Comparing the changes in the intervention group with the changes in the control group (Table 4, third column with Ratio Odds Ratio) revealed statistically significant differences at a 0.05 level in four of the questions.

#### Knowledge about user participation

For knowledge about user participation questions there was a significant difference on question no. 13 “Does your unit sponsor events/forums that educate consumers about their rights and entitlements?” (ROR = 6.5, CI = 2.8–14.9, p<0.001). In the intervention hospital significantly more professionals answered “yes” after four years.

There was a trend for difference in two questions; No. 11 “Are users invited to participate in staff training meetings at your unit?” (ROR = 5.2, CI = 0.9–30.6, p = 0.066) and no. 21 “Does the unit have a users’ committee?” (ROR = 2.3, CI = 1.0–5.2, p = 0.057). In both questions more professionals in the intervention hospitals answered “yes” after four years.

#### Practice of user participation

For the practice with user participation questions we found significant differences in two questions; No. 4 “Are clients informed about the facts about confidentiality and privacy regarding information contained in their records?” (ROR = 4.8, CI = 1.4–15.9, p = 0.012) and no. 26 “In your opinion, are next of kin in general sufficiently involved?” (ROR = 0.3, CI = 0.1–0.8, p = 0.016). For the question no. 4 significantly more of the professionals at the intervention hospital after four years answered that clients usually or always was informed. For question no. 26 significantly more professionals at the control hospitals answered yes after four years.

#### Attitudes towards user participation

For the questions regarding attitudes towards user participation we found a significant difference on one question; no. 20 “In your opinion, how would the service develop if users were involved in planning and/or carrying out the mental health service?” for the answer “Downgrading of the services and delivery” (ROR = 24.2, CI = 1.1–546.4, p = 0.045). Significantly more professionals at the intervention hospital answered yes after four years.

There was a trend for difference on question no. 18 “In your opinion, what are the most important reasons when users of mental health care don’t want to be involved?” for the answer “Lacking in ability or knowledge” (ROR = 2.8, CI = 0.8–9.2, p = 0.093). More professionals at the intervention hospital gave this as a reason after four years.

## Discussion

Professionals’ at the intervention hospital reported significantly more that the hospital sponsored events that educate consumers and that patients were informed about confidentiality, while at the control hospital a higher proportion of the professional reported that next of kin in were sufficiently involved. A higher proportion of professionals in the intervention hospital reported that involving users in planning and/or carrying out the mental health service would downgrade of the services and delivery. Overall there were more changes after four years (this study) compared to after 16 months [40].

### Strengths and limitations

This study is one of very few investigating the long term effect on professionals from user involvement. It is also one of very few controlled studies investigating the effect on professionals from an extensive and comprehensive development plan intended to enhance users’ participation in a health care organisation. These are major strengths of the study.

One very important limitation was that the samples were not matched as we wanted to ensure the participants’ anonymity. This means that the four samples could be very different and this might have influenced the findings. The results is important for these hospitals but cannot be generalized to professionals in other hospital trust or other parts of the country.

The development plan was intended to enhance user involvement across the whole organisation. We therefore included all employees in this study, regardless of whether they worked directly with patients or not. This might constitute an important limitation, since we might expect different experiences with user involvement when working in close contact with patients than when working in administrational settings. The total sample was large and the sample was representative for the gender distribution among those invited to participate. In addition, the distribution of occupational groups was similar to professionals in mental health hospitals in Central Norway [44].

As discussed in the previous paper from this study [40], the questionnaire used in this study has not been adequately tested or validated. The questionnaire has previously been used in cross-sectional studies [42,45–48] and the sensitivity of the questionnaire to measure changes over time is not known. Neither have we tested or validated the grouping of the single questions into the three themes; knowledge, practice, and attitudes. To ease the presentation of the results and the comparison of the results from this study with the previously published study after 16 months, we chose to keep the grouping. The low response rate (43%) is a limitation, but was similar to other studies on professionals in mental health services [16,36,42,45,46,48,49]. A response rate of 37 and 31% four years after the intervention is disappointing, but not surprising since the time span is long. The lower response rate at follow-up can also be attributed to not sending a reminder. It is interesting that the response rate at baseline was higher in the control hospitals (60%) than in the intervention hospital (48%). It would be reasonable to assume that the hospital, where a strategy for user development was implemented, also had professionals who were interested in answering questionnaires related to this theme.

### Time might be important to detect change

Compared to the results 16 months after the implementation [40], the results from the present study showed more effect from the development plan. Sixteen months after implementation there were no significant differences between the intervention hospital and the control hospitals and thus no significant effects from the development plan.

The results in the present study thus support the argument that organisational changes take time and confirm our assumption in the previous paper; that 16 months might not be enough time for the changes to result in any measurable effects. Although some authors have argued that organisational changes not necessarily need a lot of time to influence daily practice [50], others have argued that changing health service organisations might take considerable amounts of time before any changes have settled [18]. Several authors have stated that outcomes from user participation initiatives are dependent on how the implementation is conducted [51] and who initiated the initiative [28]. In a study exploring the implementation of user participation in different health care settings, Armstrong and colleagues outlined the following tips for successful involvement; a clear rationale for patient involvement, using the right model to achieve the desired outcomes, clarity regarding roles and responsibilities for patients, and ensuring that involvement is meaningful [52]. In our qualitative study exploring the professionals’ and user representatives’ experiences with the implementation of the development in the intervention hospital [41] we found that the professionals experiences of the implementation process depended on their position in the organisation during the process. Some described the implementation as a success and that the organization had embraced the concept of user participation. Most of those who described this had been central in the implementation processes. Those who had been less central in the implementation described on the other hand that the implementation had limited impact on the organisation’s work. This means that changes are dependent on various factors, such as responsibility and decision-making, and that the criteria for success regarding user participation often are unclear.

Multiple tests increase the risk of bias. Since the outcome measurements in this study include many questions and the analyses thus encompass many tests, the results should be interpreted with caution. We have focused on the overall direction of change in the discussion of the results.

### Interpretation of findings

Regarding knowledge on user participation the results showed that more professionals knew that users were involved in organisational settings (in educational forums and in users’ committees) after four years. The within-group changes in the intervention group confirmed an improvement on several areas of knowledge four years after implementation. These findings indicate that the increased focus on user participation due to the development plan had influenced the knowledge level among the professionals at the intervention hospital. There was no similar improvement on knowledge in the control group.

Compared to the control hospitals, there was also significantly more increase in the intervention hospital of professionals reporting that they informed clients that the information in their treatment records were confidential (question no. 4). This difference was confirmed by a significant increase within the intervention group and a non-significant decrease within the control group. This indicates that the development plan had increased the professionals’ attention on informing the service users about confidentiality. Difference was however not found in a similar question; “Are clients told they have a right to see and/or correct their records?” (No. 3) An increased attention on informing patients about their rights should be reflected also in this question. It might be that informing the patients about confidentiality and thereby building trust and alliance is easier than informing the patients that they have the right to read and correct treatment records made by the professionals.

A significant difference between the two study groups was also found on the question “In your opinion, are next of kin in general sufficiently involved?” (no. 26). A significant increase in the proportion of professionals answering “yes” was found in the control group, resulting in significantly more increase in the control group than in the intervention group. It might be that the increase in the control group reflected that the control hospitals have improved in involving the patients’ next of kin. Looking at Table 3 we see that the proportion of professionals in the control hospital who reported adequate involvement had doubled during four years, from 25 to 53%.

Although there were no differences between the groups, on the question “Do you have enough time to ensure users’ participation?” (no. 25) there were a significant increase in the proportion of professionals who answered that they had enough time to ensure user involvement in both hospitals. This implies a stronger overall focus on user involvement in the mental health services and a stronger feeling of having enough time for this work. This is in line with the results from the qualitative study exploring the implementation process, where the professionals reported that the hospital in general had increased the focus on user involvement [41].

Although a very large proportion of the professionals reported that clients should be involved in the planning of their own treatment and in the evaluation of their presenting problems, it was a surprising finding that professionals in the intervention hospital more frequently reported that it would lead to a downgrading in the services if users took part in planning and conduct of the services. This is also contrary to previous research that has indicated that attitudes towards involvement of users can be improved after having experienced user involvement in practice [15,53] and that those positive towards user involvement have previous experience from user participation [28].

## Conclusions

This non-randomised controlled study is the first to investigate the long term effect on mental health professionals four years after implementing an organisational development plan to enhance user participation. The plan led to some significant improvements in professionals’ knowledge and practice of user participation. As there was more effect after four years than after 16 months, this study indicates that it takes long time before the effect of complex interventions to enhance patient participation in organisations can be detected among the professionals.

### Clinical and research implications

More long-term studies are needed to build further knowledge on the effect on professionals from implementing complex initiatives to enhance user participation in health care organisations. Robust instruments to measure the development of professionals’ knowledge practice and attitudes towards user involvement are needed. In clinical work it is important to allow for enough time and patience for complex interventions to make their mark on the professionals and their practice.

## Supporting Information

S1 Dataset Minimal dataset underlying the findings. (SAV)
